# Dynamics of a diffusive model for cancer stem cells with time delay in microRNA-differentiated cancer cell interactions and radiotherapy effects

**DOI:** 10.1038/s41598-024-55212-4

**Published:** 2024-03-04

**Authors:** Frank Eric Essongo, Alain Mvogo, Germain Hubert Ben-Bolie

**Affiliations:** 1 Laboratory of Nuclear Physics, Dosimetry and Radiation Protection, Department of Physics, Faculty of Science, University of Yaounde I, P.O. Box 812, Yaounde, Cameroon; 2 Laboratory of Biophysics, Department of Physics, Faculty of Science, University of Yaounde I, P.O. Box 812 Yaounde, Cameroon

**Keywords:** Cancer cells, Cancer stem cells, MicroRNAs, Hopf bifurcation, Time delay, Circular patterns, Radiotherapy, Biophysics, Cancer, Mathematics and computing, Biological physics, Biophysics, Cancer, Mathematics and computing, Biological physics

## Abstract

Understand the dynamics of cancer stem cells (CSCs), prevent the non-recurrence of cancers and develop therapeutic strategies to destroy both cancer cells and CSCs remain a challenge topic. In this paper, we study both analytically and numerically the dynamics of CSCs under radiotherapy effects. The dynamical model takes into account the diffusion of cells, the de-differentiation (or plasticity) mechanism of differentiated cancer cells (DCs) and the time delay on the interaction between microRNAs molecules (microRNAs) with DCs. The stability of the model system is studied by using a Hopf bifurcation analysis. We mainly investigate on the critical time delay $\tau _{c}$, that represents the time for DCs to transform into CSCs after the interaction of microRNAs with DCs. Using the system parameters, we calculate the value of $\tau _{c}$ for prostate, lung and breast cancers. To confirm the analytical predictions, the numerical simulations are performed and show the formation of spatiotemporal circular patterns. Such patterns have been found as promising diagnostic and therapeutic value in management of cancer and various diseases. The radiotherapy is applied in the particular case of prostate model. We calculate the optimum dose of radiation and determine the probability of avoiding local cancer recurrence after radiotherapy treatment. We find numerically a complete eradication of patterns when the radiotherapy is applied before a time $t < \tau _{c}$. This scenario induces microRNAs to act as suppressors as experimentally observed in prostate cancer. The results obtained in this paper will provide a better concept for the clinicians and oncologists to understand the complex dynamics of CSCs and to design more efficacious therapeutic strategies to prevent the non-recurrence of cancers.

## Introduction

Cancer cells result of abnormal growth and uncontrolled division of normal cells^1^. Cancers grow from stem cells in the way that healthy organs do. The discovery of the existence of cancer stem cells (CSCs) in cancers has led to an understanding the mechanisms of cancer proliferation and development. The CSCs have been identified and isolated in many so-called solid tumors such as breast cancer, pancreatic cancer, glioblastoma (a type of brain cancer) and colon adenocarcinoma^2^. Recent reports, primarily for haematopoietic malignancies, have suggested that CSCs can be selectively targeted without ablating normal stem cell function. The CSCs can be more resistant to chemotherapy and radiotherapy^3^. Sometimes, after cancer treatment, the cancer comes back or recurs, this is called a cancer recurrence. The CSCs are a minor population of tumor cells that possess the stem cell property of self-renewal. Dysregulation of stem cell self-renewal is likely requirement for the development of cancer^4^. The CSCs are biologically unique cells within a tumor, possessing self renewal capacity and produce progeny with self proliferative capacity, similar to normal stem cells^5^. All these properties have led many researchers to believe that CSCs are responsible for the recurrence and spread of glioblastoma and other cancers, since they give rise to multiple other cancer cells and produce new tumors^6^.

MicroRNA molecules (MicroRNAs) are small non-coding RNA molecules that regulate gene expression by binding to RNA messenger, thereby preventing its translation into proteins^7^. The microRNAs are key regulators of the activity of CSCs and are also involved in the transformation of differentiated cancer cells (DCs) into CSCs^8,9^. The DCs are tumor cells that undergo a process of differentiation, meaning they acquire specific characteristics to particular type of tissue. The microRNAs play an important role in many biological processes, including development, cell differentiation, and tumor progression. Similarly, complex cellular secretions (serum, plasma, urine, saliva, blood) are regulated by microRNAs in extracellular environment. The microRNAs are used as powerful biomarkers^10–12^, of various human pathologies precisely when measured in the bloodstream. For example, the detection of abnormal levels of microRNAs in the blood of cancer patients makes them a very promising diagnostic tool in cancerology. In adrenocortical carcinomas or adrenocorticalomas, miRNAs are used as non-invasive molecular markers for prognostic evaluation within a very short time after surgery. It has been shown in^13^ that microRNA-483-5p is significantly more abundant in the serum of patients who have recurred within 3 years after surgery compared to those who have not recurred. Some researchers have found that a specific microRNA can act as an oncogene in some types of cancer while the same microRNA can also act as a cancer cell suppressor in other cancers. For example, the microRNA-29 molecule acts as a tumour suppressor in lung tumours and as an oncogene in breast cancer^14,15^.

The CSCs are responsible to the tumor relapse, and their dynamics are closely linked to disease progression. Therefore, there is an urgent need for improved diagnostic methods that provide more precise clinical assessments and sensitive detection of symptoms at earlier stage of the disease. In this context, mathematical modeling has been successfully applied. For example, Bonnet and Dick^16^ have shown in a mathematical model that acute myeloid leukemia is indeed organized as a hierarchy, with the disease originating from a primitive hematopoietic cell. Wang et al.^17^ have studied network-based methods for the identification of microRNA-target pairs in cancer. A long the same line, Konstorum et al.^18^ demonstrated that feedback regulation in a model for CSCs can cause an allee effect. On this background, Olmeda and Amar^19^ investigated clonal pattern dynamics in the concept of CSCs and showed that disordered patterns can be obtained inside a stable growing contour driven by the cancer stem cells. Very recently, Mori and Amar^20^ studied the importance of stochasticity and drug effects in a dynamical model for CSCs. These above-mentioned works implicitly assume that biological transitions such as the interactions between DCs or CSCs with signaling molecules and proteins are instantaneous. However, in reality these take time. Time delays play an important role in the modeling of life phenomena in various fields of applications^21–23^. Time delays are often associated with complex molecular mechanisms, such as the activation of specific signaling pathways or the regulation of gene expression by microRNAs.

   In this paper, we investigate a diffusive model for tumor initiation composed of CSCs, DCs and other cells that do not contribute significantly to the dynamics of the tumor, such as dead, quiescent and healthy cells. The dynamical system is based on the proliferation rates controlled by chemical pathways and physical interaction for each cell population. It mainly takes into account the diffusion of cells, the de-differentiation (or plasticity) mechanism of differentiated cancer cells (DCs) and the time delay on molecule-cell interaction of microRNAs with DCs. We propose an efficiency mathematical approach that includes the radiotherapy effects. Radiotherapy is one of the most commonly used treatments for cancer, and its effectiveness largely depends on the sensitivity of cancer cells to radiation^24^. We show that the radiotherapy can applied before a certain critical time delay in the dynamical system for a complete eradication of the tumor. This critical time delay represents the time from which some DCs transform into CSCs. The rest of the paper is organized as follows. In “The diffusive model with time delay and linear stability analysis” section, we present the model formulation and perform the linear stability analysis. We calculate the value of critical time delay for 3 different cancer models, namely prostate, lung and breast cancers. In “Analytical and numerical results” section, we integrate the radiotherapy function in the model and some analytical and numerical results are presented and discussed. “Discussion and conclusion” section concludes the paper.

## The diffusive model with time delay and linear stability analysis

### Model formulation

There are a number of mathematical models used to describe the dynamics of cancer stem cells (CSCs). These models include the Dick’s model^16^, the Reya’s model^25^ and many others^26–28^. The model investigated in this paper is the one proposed by Konstorum et al.^18^ and improved by Olmeda and Amar^19^ and Mori and Amar^20^. The mathematical model considers the dynamics of three types of cells: CSCs with similar characteristics as normal stem cells or progenitor cells such as self-renewal ability and multi-lineage differentiation to drive tumor growth and heterogeneity, differentiated cancer cells (DCs) which are specific to the tumor and vary across organs and other cells (C) or inert constituents. The CSCs are the sub-populations that undergo cell division leading for the proliferation of cells. The model also considers eliminating intermediate progenitors to the proliferation process. A CSC can divide symmetrically into two CSCs or two DCs, or asymmetrically into one CSC and one DC. The average number of CSCs generated in division is regulated by an activator Wnt-$\beta$ (Wingless-related integration site) catenin pathway proteins^8^ with concentration *a*. Experimental evidence shows that a feedback mechanism exists, triggered by phenotypic changes of DCs that revert to the CSC state. This process, also known as cancer cell plasticity, is driven by an activator microRNA molecules (microRNAs) with concentration *m*^9^. It is now proved that differential dynamical systems with time delay and diffusion play an important role in the modeling of real life phenomena in biology, physics and medicine^21–23,29–31^. Also, the modeling of CSCs with diffusion has the potential to yield invaluable insights into cancer metastasis, disease progression, and treatment efficacy assessment^32^. Then, to improve the model, we include the diffusion process in the dynamical equations of all types of cells and molecules. More interesting, we include a time delay $\tau$ in the dynamical equation of CSCs to represent the time needed for DCs to produce new CSCs after the interaction of microRNAs with DCs. All these assumptions can be explained in the following set of equations:

1 $$\begin{aligned} \left\{ \begin{aligned} \frac{d S}{d t}&=(2p(D,a)-1)\varepsilon S + q(m(t-\tau ))D(t-\tau )+D_{S}\Delta S,\\ \frac{d D}{d t}&=2(1-p(D,a))\varepsilon S - (d+q(m))D+D_{D}\Delta D,\\ \frac{d a}{d t}&=a(\beta S\frac{a}{1+a} - \alpha )+D_{a}\Delta a,\\ \frac{d m}{d t}&=\gamma e^{ -S/S_{0}} - \alpha m+D_{m}\Delta m, \end{aligned} \right. \end{aligned}$$

where $S=S(x, y, t)$ is the concentration of CSCs, $D=D(x, y, t)$ is the concentration of DCs, $a=a(x, y, t)$ represents the concentration of Wnt-$\beta$ catenin pathway proteins and $m=m(x, y, t)$ the concentration of microRNAs. The quantities 2*p*(*D*, *a*) denotes the probability to gain two CSCs per mitosis and $q(m(t-\tau ))$ represents the DCs dedifferentiation rate. They satisfy:

2 $$\begin{aligned} p(D,a) =\frac{\eta a}{(1+\eta a)(1+\psi D)},\quad q(m(t-\tau )) =\frac{q_{0}}{2}\biggl (1+\tanh \biggl (\dfrac{m(t-\tau )-m_{0}}{\sigma }\biggr )\biggr ), \end{aligned}$$

where the parameter $q_0$ represents the maximum conversion rate per year of DCs into CSCs and $m_0$ the tiny concentration of microRNAs. The parameters $\alpha , \beta$ respectively represent the degradation rate of proteins, controlling the aggressiveness of self-renewal Wnt-$\beta$ catenin pathway proteins. The parameter $\sigma$ measures the sensitivity of CSCs when interacting with microRNAs. The parameter $\eta$ refers to the definition of a probability coefficient *p*(*D*, *a*) for the inhibitor produced by *D* cells, where $\psi$ gives the intensity of the brake. The parameter *d* is the differentiated cancer cells death rate ($d>0$). The parameter $\varepsilon$ represents a time unit the inverse of the mitotic rate of the CSCs. The quantity $S_{0}$ is a tiny fraction of *S* of the entire tumor population. In practice when *S* is below $S_{0}$, the activator *m* starts to grow as *q*(*m*) , leadind to an increase of *S*. Indeed, when *m* stimulates DCs that possess plasticity, the DCs transform into CSCs over a period of time. Given this process, we have considered a time delay $\tau$ which represents the time needed for DCs to produce CSCs after the interaction with microRNAs. In practice, time delays in biological systems may be large^33^. The term $\Delta$ is the Laplacian operator in two dimensions, such that $\Delta =\frac{\partial ^{2}}{\partial x^{2}}+\frac{\partial ^{2}}{\partial y^{2}}$.

From biological considerations, we assume: $S\ge 0, D\ge 0, a\ge 0, m\ge 0$, $(x, y) \in \Sigma = (0, L)\times (0, L )$ and we use zero-flux boundary conditions: $\frac{\partial S}{\partial \varpi }\mid _{\partial \Sigma }$=$\frac{\partial D}{\partial \varpi }\mid _{\partial \Sigma }$=$\frac{\partial a}{\partial \varpi }\mid _{\partial \Sigma }$=$\frac{\partial m}{\partial \varpi }\mid _{\partial \Sigma }$=0. The main reason for choosing such boundary conditions is that we are interested in the self-organization of pattern and zero-flux conditions imply no external input. The length of $x, y \in (0, L)$ is measured in millimeter [mm], where the length scale *L* denotes the size of the system in square domain and $\varpi$ is the outward unit normal vector of the boundary $\partial \Sigma$. In practice, the length scale *L* is the average penetration depth of cells in tissue. The time is measured in years. The units of variables and parameters used in this paper are given in the Table 1.

**Table 1:** Variables and parameters of the model.

Variable	Description	Unit adopted	
*S*	Concentration of CSCs	$\mathrm{cell.mm}^{-3}$	
*D*	Concentration of DCs	$\mathrm{cell.mm}^{-3}$	
*a*	Concentration of Wnt-$\beta$	$\mathrm{molecule.mm}^{-3}$	
*m*	Concentration of microRNAs	$\mathrm{molecule.mm}^{-3}$	

Parameter	Description	Value $\&$ Unit	References
$\eta$	Positive feedback strength of Wnt-$\beta$	1 $\textrm{mm}^{3}.\textrm{molecule}^{-1}$	Modified from^19^
$\psi$	Negative feedback strength of DCs	0.5 $\textrm{mm}^{3}.\textrm{cell}^{-1}$	Modified from^34^
$\varepsilon$	Mitotic rate of the CSCs	1 $\textrm{year}^{-1}$	Modified from^20^
*q*	DCs dedifferentiation rate	$\textrm{year}^{-1}$	^19^
$q_{0}$	Maximum DCs dedifferentiation rate	q_0_ $>$d $\textrm{year}^{-1}$	^19^
$m_{0}$	Minimal concentration of microRNAs	0.05 $\mathrm{molecule.mm}^{-3}$	Modified from^19^
*d*	DCs death rate	36.5–109.5 $\textrm{year}^{-1}$	^20^
$\alpha$	Degradation rate of proteins or molecules	0.3 $\textrm{mm}^{3}.\textrm{molecule}^{-1}$	Modified from^35^
$\beta$	Aggressiveness of self-renewal Wnt-$\beta$ rate	1 $\textrm{mm}^{3}.\textrm{cell}^{-1}.\textrm{year}^{-1}$	Modified from^35^
$\gamma$	Maximum concentration of microRNAs	1 $\mathrm{molecule.mm}^{-3}.\textrm{year}^{-1}$	Modified from^19^
$\sigma$	Sensitivity of CSCs when interacting with microRNAs	0.05 $\mathrm{molecule.mm}^{-3}$	Modified from^19^
$S_{0}$	Minimal concentration of CSCs	0.038 $\mathrm{cell.mm}^{-3}$	Modified from^19^
$D_{S}$	Diffusion coefficient of CSCs	0.031536 $\textrm{mm}^{2}.\textrm{year}^{-1}$	^36^
$D_{D}$	Diffusion coefficient of DCs	0.031536 $\textrm{mm}^{2}.\textrm{year}^{-1}$	^36^
$D_{a}$	Diffusion coefficient of Wnt-$\beta$	0.365 $\textrm{mm}^{2}.\textrm{year}^{-1}$	^13^
$D_{m}$	Diffusion coefficient of microRNAs	0.365 $\textrm{mm}^{2}.\textrm{year}^{-1}$	^13^

### Linear stability analysis

The linear stability analysis is attributed to the highest sensibility of biological system^37^. The linear stability analysis deals with infinitesimal perturbation and consists to perturb the steady state of the system. It is easy to check that system (1) has three equilibrium points, $E^{(1)}=(0,0,0,m_{1})$, $E^{(2)}=(S_{2},D_{2},0,m_{2})$ and $E^{(3)}=(S_{3},D_{3},a_{3},m_{3})$.

The first steady state of system (1) is $E^{(1)}=(0,0,0,m_{1})$, that is, $S=0,D=0, a=0$ and $m_{1}$ an arbitrary value. Notice that the equilibrium $E^{(1)}$ corresponds to the hypothetical situation where there is no CSCs, no DCs and the $Wnt-\beta$ protein is inactive.

The second steady state is $E^{(2)}=(S_{2},D_{2},0,m_{2})$, that is, $S_{2}=-S_{0}Log\biggl (\frac{\alpha m_{2}}{\gamma }\biggr ), D_{2}=\frac{S_{2}}{d}, a_{2}=0$ and $m_{2}$ an arbitrary value. $E^{(2)}$ is biologically meaningful and $E^{(2)}$ corresponds to the situation where Wnt-$\beta$ protein is inactive and the microRNAs has arbitrary values. This equilibrium point is always stable if $d<q_{0}$ and $m_{2}<\frac{\gamma }{\alpha }$.

The third steady state $E^{(3)}=(S_{3},D_{3},a_{3},m_{3})$, that is, $S_{3}=\frac{\alpha (1+a_{3})}{\beta a_{3} }, D_{3}=\frac{\alpha (1+a_{3})}{\beta d a_{3}}, m_{3}=\frac{\gamma }{\alpha } e^{-S_{3}/S_{0}}$ and $a_{3}=\frac{1+{\frac{\psi \alpha }{d\beta }}(1+\eta )}{2\eta (1-{\frac{\psi \alpha }{d\beta }})} \left( 1+\sqrt{1+\frac{4{\frac{\psi \alpha }{d\beta }}\eta (1-{\frac{\psi \alpha }{d\beta }})}{(1+{\frac{\psi \alpha }{d\beta }}(1+\eta ))^{2}}}\right) .$ $E^{3}$ is an unstable equilibrium point. Biologically, $E^{3}$ corresponds to the case where the wnt-$\beta$ catenin signaling pathway and microRNAs are activated, leading to uncontrolled proliferation of cancer stem cells. This also can be related to the chronic phase of the disease. Our goal is to study the phenomenon of conversion of differentiated cancer cells into cancer stem cells influenced by microRNAs when the wnt-$\beta$ signaling pathway is not activated. At this stage, cancer proliferation can be controlled and the patient can be treated if appropriate therapeutic strategies are used. In the rest of the paper, for the biomedical view point, we will investigate the dynamics of the system at the stable equilibrium point $E^{(2)}$.

We now explore the dynamics of diffusion-driven instability with respect to $E^{(2)}$. For this, consider small spatiotemporal perturbations $\delta S(x, y, t)$, $\delta D(x, y, t)$, $\delta a(x, y, t)$ and $\delta m(x, y, t)$ on homogenous stady state $E^{(2)}=(S_{2}, D_{2}, 0, m_{2})$ so that we have:

3 $$\begin{aligned} \begin{aligned} S(x, y, t)&=S_{2}+ \delta S(x, y, t); \;\ D(x, y, t)=D_{2}+ \delta D(x, y, t),\\ a(x, y, t)&=0+ \delta a(x, y, t); \;\ m(x, y, t)=m_{2}+ \delta m(x, y, t). \end{aligned} \end{aligned}$$

Expressing spatiotemporal perturbations in the form:

4 $$\begin{aligned} \begin{aligned} \delta S(x, y, t)=\delta S_{2}e^{\lambda t}cosk_{x}xcosk_{y}y; \;\ \delta D(x, y, t)=\delta D_{2}e^{\lambda t}cosk_{x}xcosk_{y}y,\\ \delta a(x, y, t)=\delta a_{2}e^{\lambda t}cosk_{x}xcosk_{y}y; \;\ \delta m(x, y, t)=\delta m_{2}e^{\lambda t}cosk_{x}xcosk_{y}y, \end{aligned} \end{aligned}$$

where $\lambda$ is the growth rate of the perturbation in time *t*. The parameters $\delta S_{2}$, $\delta D_{2}$, $\delta a_{2}$ and $\delta m_{2}$ represent the amplitudes, and $k_{x}$ and $k_{y}$ are the wavenumbers in the *x* and *y* directions, respectively. Inserting Eqs. (3) and (4), we obtain the following matrix equation for eigenvalues

$$\begin{aligned} \begin{pmatrix} -1-D_{S}k^{2}-\lambda &{}\quad de^{-\lambda \tau } &{}\quad \frac{2S_{2}\eta }{1+\psi D_{2}} &{}\quad \frac{2dD_{2}}{\sigma }\biggl (1-\frac{d}{q_{0}}\biggr )e^{-\lambda \tau }\\ 2 &{}\quad -2d-D_{D}k^{2}-\lambda &{}\quad -\frac{2S_{2}\eta }{1+\psi D_{2}} &{}\quad -\frac{2dD_{2}}{\sigma }\biggl (1-\frac{d}{q_{0}}\biggr ) \\ 0 &{}\quad 0 &{}\quad -\alpha -D_{a}k^{2}-\lambda &{}\quad 0 \\ -\frac{\alpha }{S_{0}}m_{2} &{}\quad 0 &{}\quad 0 &{}\quad -\alpha -D_{m}k^{2}-\lambda \\ \end{pmatrix} =0. \end{aligned}$$

The eigenvalues $\lambda$ are the solutions of the quadratic equation as follows:

5 $$\lambda _{k}^{4} + C_{11}\lambda _{k}^{3}+C_{22}\lambda _{k}^{2}+C_{33}\lambda _{k}+C_{44} + (E_{11}\lambda _{k}^{2}+E_{22}\lambda _{k}+E_{33} )e^{-\lambda _{k} \tau }=0,$$

where the parameters $C_{11}, C_{22}, C_{33}, C_{44}, E_{11}, E_{22}$ and $E_{33}$ are given in Online Appendix.

Let $i\omega _{k}$ (where $\omega _{k}$ is positive) be the root of Eq. (5) and separating the real and imaginary parts, we obtain the following system of equations:

6 $$\left\{ {\begin{array}{*{20}l} {C_{{22}} \omega _{k}^{2} - C_{{44}} - \omega _{k}^{4} = E_{{22}} \omega _{k} \sin \omega _{k} \tau + (E_{{33}} - E_{{11}} \omega _{k}^{2} )\cos \omega _{k} \tau ,} \hfill \\ {C_{{33}} \omega _{k} - C_{{11}} \omega _{k}^{3} = (E_{{33}} - E_{{11}} \omega _{k}^{2} )\sin \omega _{k} \tau - E_{{22}} \omega _{k} \cos \omega _{k} \tau .} \hfill \\ \end{array} } \right.$$

From Eq. (6), we obtain

7 $$\begin{aligned} \left\{ \begin{aligned} \cos \omega _{k}\tau&=\dfrac{(E_{33}-E_{11}\omega ^{2}_{k})(C_{22}\omega ^{2}_{k}-C_{44}-\omega ^{4}_{k})-E_{22} \omega _{k}(C_{33}\omega _{k}-C_{11}\omega ^{3}_{k})}{(E_{33}-E_{11}\omega ^{2}_{k})^{2}+E_{22}^{2}\omega ^{2}_{k}}, \\ \sin \omega _{k}\tau&=\dfrac{E_{22}\omega _{k}(C_{22}\omega ^{2}_{k}-C_{44}-\omega ^{4}_{k}) +(E_{33}-E_{11}\omega ^{2}_{k})(C_{11}\omega ^{3}_{k}-C_{33}\omega _{k})}{(E_{33}-E_{11} \omega ^{2}_{k})^{2}+E_{22}^{2}\omega ^{2}_{k}}, \end{aligned} \right. \end{aligned}$$

which leads to

8 $$\begin{aligned} \omega _{k}^{8}+(C_{11}^{2}-2C_{22})\omega _{k}^{6}+(C_{22}^{2}+2C_{44}-2C_{11}C_{33} -E_{11}^{2})\omega _{k}^{4}+(C_{33}^{2}+2E_{11}E_{33}-2C_{22}C_{44}-E_{22}^{2})\omega _{k}^{2} +(C_{44}^{2}-E_{33}^{2})=0. \end{aligned}$$

We now suppose that (8) has at least one positive root. Then, (8) has a positive root $\omega _{k}=\omega ^{*}$ such that (5) has a pair of purely imaginary roots $\mp i\omega _{k}$. Then, we can obtain the corresponding critical value of the delay for $\omega _{k}$

9 $$\begin{aligned} \tau ^{(j)}_{k} = \left\{ \begin{aligned}&\frac{1}{\omega _{k}}arctan\biggl (\frac{Z_{11}^{k}}{Z_{22}^{k}}\biggl )+\frac{j2\pi }{\omega _{k}},\quad if\quad Z_{11}^{k}>0 \\&\frac{1}{\omega _{k}}\left[ \pi +arctan\biggl (\frac{Z_{11}^{k}}{Z_{22}^{k}}\biggl )\right] +\frac{j2\pi }{\omega _{k}},\quad if\quad Z_{22}^{k}<0 \\&\frac{1}{\omega _{k}}\left[ 2\pi +arctan\biggl (\frac{Z_{11}^{k}}{Z_{22}^{k}}\biggl )\right] +\frac{j2\pi }{\omega _{k}},\quad if\quad Z_{11}^{k}<0,Z_{22}^{k}>0, \end{aligned} \right. \end{aligned}$$

where $\quad j=0,1,2...$ from (12), $Z_{11}^{k}=E_{22}\omega _{k}(C_{22}\omega _{k}^{2}-C_{44}-\omega _{k}^{4}) +(E_{33}-E_{11}\omega _{k}^{2})(C_{11}\omega _{k}^{3}-C_{33}\omega _{k})$ and $Z_{22}^{k}=(E_{33}-E_{11}\omega _{k}^{2})(C_{22}\omega _{k}^{2}-C_{44} -\omega _{k}^{4})-E_{22}\omega _{k}(C_{33}\omega _{k}-C_{11}\omega _{k}^{3})$.

Differentiating both sides of Eq. (8) with respect to $\tau$, we obtain

$$\begin{aligned} \biggl [\dfrac{d\lambda _{k}}{d\tau }\biggl ]^{-1}=\dfrac{4\lambda ^{3}_{k} +3C_{11}\lambda ^{2}_{k}+2C_{22}{\lambda _{k}}+C_{33}+(2E_{11}\lambda _{k} +E_{22})e^{-\lambda _{k}\tau }}{{\lambda _{k}}(E_{11}\lambda ^{2}_{k}+E_{22}\lambda _{k}+E_{33})e^{-\lambda _{k}\tau }} - \dfrac{\tau }{\lambda _{k}}, \end{aligned}$$

which yields, at $\tau =\tau _{c}=\tau ^{(j)}_{k}$, the following transversality condition :

10 $$\begin{aligned} sign Re\biggl [\dfrac{d\lambda _{k}}{d\tau }\biggl ]_{\tau =\tau _{c}}= Re\biggl [\dfrac{d\lambda _{k}}{d\tau }\biggl ]^{-1}_{\tau =\tau _{c}}= sign\biggl (\dfrac{{\tilde{A}}_{R}{\tilde{B}}_{R}+{\tilde{A}}_{I}{\tilde{B}}_{I}}{{\tilde{B}}_{R}^{2}+{\tilde{B}}_{I}^{2}}\biggr )>0, \end{aligned}$$

where

11 $$\begin{aligned} \left\{ \begin{aligned} {\tilde{B}}_{R}&={-E_{22}\omega ^{2}\cos \omega \tau _{c}+(-E_{11}\omega ^{3}+E_{33}\omega )\sin \omega \tau _{c}},\\ {\tilde{B}}_{I}&={(E_{33}\omega -E_{11}\omega ^{3})\cos \omega \tau _{c}+E_{22}\omega ^{2}\sin \omega \tau _{c}},\\ {\tilde{A}}_{R}&=E_{22}\cos \omega \tau _{c}- 3C_{11}\omega ^{2}{+2E_{11}\omega \sin \omega \tau _{c} +C_{33}},\\ {\tilde{A}}_{I}&=-4\omega ^{3}{+2E_{11}\omega \cos \omega \tau _{c}+2C_{22}\omega -E_{22}sin\omega \tau _{c}}. \end{aligned} \right. \end{aligned}$$

The transversality condition is a mathematical requirement to ensure the validity of the model. In the case of the model under study, the transversality condition must be greater than zero. This means that the critical time delay, which represents the time delay after which a differentiated cancer cell converts to a cancer stem cell, must be strictly positive. We recall that when $\tau =0$ equilibrium $E^{(2)}$ is always stable when ($d<q_{0}$) and $(m_{2}<\frac{\gamma }{\alpha })$.

From, the transversality condition (10) for $\tau >\tau _{c}$, $E^{(2)}$ is unstable and stable for $\tau <\tau _{c}$.

When $\tau = \tau _{c}$, Hopf bifurcation occurs.

## Analytical and numerical results

### Analytical results

In this section, we present some analytical results related with this work. We calculate the value of the critical time delay $\tau _{c}$ given in Eq. (9) for some cancers, namely prostate, lung and breast cancers. The estimated values of $q_0$ and *d* that we use are related to a cancer and taken in the literature. The results are summarized in Table 2.

**Table 2:** Value of the critical time delay $\tau _{c}$ for some cancers.

Type of cancer	Values $q_{0}$ and *d*	Value of the critical time delay $\tau _{c}$
Prostate cancer	$q_{0}$ = 87.6 $\textrm{year}^{-1}$, *d* = 87.2 $\textrm{year}^{-1}$^9,20,38^	11.8964 $\textrm{years}$
Lung cancer	$q_{0}$= 109.97 $\textrm{year}^{-1}$, *d* = 80 $\textrm{year}^{-1}$^39–41^	0.6287 $\textrm{year}$
Breast cancer	$q_{0}$ = 70.39 $\textrm{year}^{-1}$, $d=$ 45 $\textrm{year}^{-1}$^42,43^	0.5858 $\textrm{year}$

We recall that the critical time delay $\tau _{c}$ represents the time for DCs to transform into CSCs after the interaction of microRNAs with DCs. At this time, the system becomes unstable. From the biological view point, this situation corresponds to the proliferation of CSCs indicating progression leading sometime to metastases^44^. The critical time delay values obtained in Table 2 can be also considered to the relapse period of cancer. For the breast cancer, $\tau _{c} = 0.5858$ year, and this value can correspond with the relapse period of triple-negative breast cancer^45^. This type of breast cancer is characterized by the absence of over-expression of hormone receptors for estrogen and progesterone and absence of over-expression of the HER2 growth factor^45^. Among the different types of breast cancer, triple-negative cancers are the ones that recur most frequently and more rapidly than other tumor types. In fact, between 6 and 24 months after the primary tumor, 52.3 percents of triple-negative metastases are discovered, 24 percents of metastases are discovered for HER2+ and 12 percent of metastases are discovered for HR+HER2-^46^. For a lung cancer $\tau _{c} = 0.6287$ is less than 2 years and this time corresponds to an early recurrence observed in small cell lung cancer^47,48^. For a prostate cancer $\tau _{c} = 11.8964$. This result confirm the one obtained by Pound et al.^49^ who have shown that a very low recurrence of prostate cancer 11 years later is possible, and that 10 percents of men with undetectable prostate-specific antigen (PSA) levels 10 years after radical prostatectomy subsequently develop biochemical recurrence, which means an increase in the PSA levels without clinical signs of cancer. Therefore, an estimation of the recurrence period of cancer can be important to adjust therapeutic treatment and to take preventive measures to reduce the risk of recurrence.

###  Numerical results

We perform extensive numerical simulations of the spatially extended model (1) in two-dimension spaces in the case of prostate cancer model parameters. The numerical simulations employ a system size of $100\times 100$ space units. The numerical integration of model (1) was performed in MATLAB by means of forward Runge-Kutta of fourth order with Hermite interpolation. This method is favored for its accuracy and stability compared to other numerical methods^50,51^. A time step $\Delta t=0.01$ and a space step of $\Delta x=\Delta y=0.2$ are used. Further, the model (1) is investigated numerically under the zero flux boundary conditions. The choice of initial value indicates small inhomogeneous spatial perturbation from homogeneous equilibrium state $\hbox {E}^{(2)}$. The initial distribution of the populations are taken as

$$\begin{array}{*{20}l} {S(x,y,0) = S_{2} + ( - 0.1cos((x^{2} + y^{2} )\pi - 0.01sin((x^{2} + y^{2} )\pi ))),} \hfill \\ {D(x,y,0) = D_{2} + ( - 0.0002cos((\sqrt {x^{2} + y^{2} } )\pi + 0.0003sin((\sqrt {x^{2} + y^{2} } )\pi ))),} \hfill \\ {a(x,y,0) = a_{2} + (0.002cos(x + 2)^{2} + 0.001sin(y + 4)^{2} ),} \hfill \\ {m(x,y,0) = m_{2} + (0.001cos(x - 2)^{2} + 0.001sin(y - 4)^{2} ),} \hfill \\ {{\text{where}}\;\{ (S_{2} ,D_{2} ,a_{2} ,m_{2} )\} = \{ (0.140000,0.001605,0.000000,0.080000)\} } \hfill \\ \end{array}$$ .

To avoid numerical stiffness, we performed the simulations qualitatively for smaller values of the step sizes.

#### Bifurcation diagrams and time series

**Figure 1:**
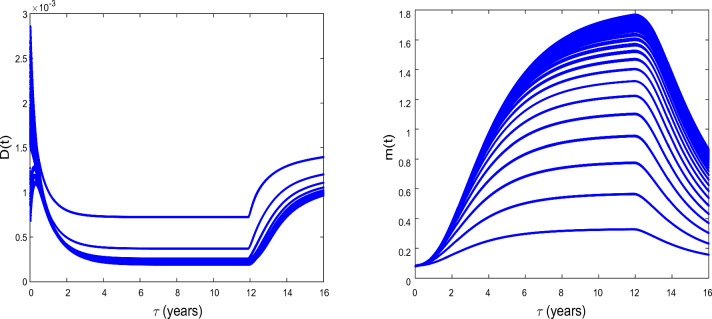
Bifurcation diagrams of *D*(*t*) and *m*(*t*) . $D_{S}=D_{D}=0.031536$ and $D_{a}=D_{m}=0.365$ and the values of the other parameters values are given in the Table 1. We observe that Hopf bifurcation occurs at $\tau =\tau _{c}= 11.8964$ for prostate cancer.

We now explore the bifurcation of the system, in order to show that the system can manifest diverse biological phenomena such as shifts in cell concentrations and cell-protein interactions. Figure 1 shows the emergence of bifurcation in the system. In this figure, the equilibrium state is represented by a stationary point where the system trajectory remains constant. Instability is indicated by the Hopf bifurcation and its occurs at the critical time delay $\tau _{c}=11.8964$. In fact, when $\tau <\tau _{c}$, the system is stable at the equilibrium $E^{(2)}$ and when $\tau >\tau _{c}$, the system becomes unstable. From the biological point of view, the instability occurs at $\tau =\tau _{c}$, that is when the conversion of DCs into CSCs takes place indicating tumor progression. It is observed an increase in the concentration *D* of DCs and a decrease in the concentration *m* of microRNAs. Thus, the critical time delay $\tau _{c}$ can be viewed as the initial time for the proliferation of CSCs. Then, Hopf bifurcation indicates the transition to the disease state, characterized by the proliferation of CSCs^25^. This bifurcation can serve as a crucial indicator of substantial disruptions in the system’s normal functioning, with potentially implications for health and disease.

**Figure 2:**
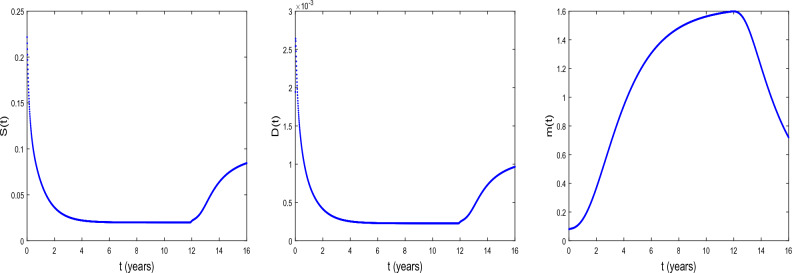
The time series of *S*(*t*), *D*(*t*), and *m*(*t*) at $\tau =\tau _{c}= 11.8964$ for $D_{S}=D_{D}=0.031536$ and $D_{a}=D_{m}=0.365$. The other parameter values are given in the Table 1.

Figure 2 shows the time series of *S*(*t*), *D*(*t*) and *m*(*t*) . We observe that before the critical time delay $\tau _{c}=11.8964$, the concentrations *S* and *D* of CSCs and of DCs, respectively decrease while the concentration *m* of microRNAs decreases as time progresses. For such a situation, it has been shown that the microRNAs can reduce tumorigenicity^7^. Some works reported also in vivo that the regulation of *NF*90 greatly reduces ovarian tumor proliferation and invasion^52^. This regulation also greatly reduces the size of the ovarian tumor and the number of metastases. We also observe a dormancy period of cancer cells. From the biomedical view point, during this period the patients often have no symptoms and the tumor can be undetectable using the usual diagnostic tools such as PET scan (nuclear medicine imaging examination). After the time $\tau _{c}=11.8964$, we observe the inverse of the scenario, the concentration *m* decreases as time progresses while the concentrations *S* and *D* increase. In this case, oncogenic role for microRNAs can be reported.

#### Pattern formation and effect of diffusion on cancer cells

The diffusion of cancer stem cells is a complex phenomenon characterized by the migration and invasion of these cells from the primary tumor to distant organs, leading to the formation of metastases^44^. This process, known as metastasis, is a major contributor to cancer-related deaths, as it confers resistance to treatment strategies and increases the risk of relapse^53^. Therefore the effect of diffusion on cancer stem cells is an important to understand cancer mechanisms and develop new therapeutic strategies. The effect of diffusion on cancer stem cells could lead also to the development of a prevention strategy for non-recurrence in either non-metastatic or metastatic cancer.

**Figure 3:**
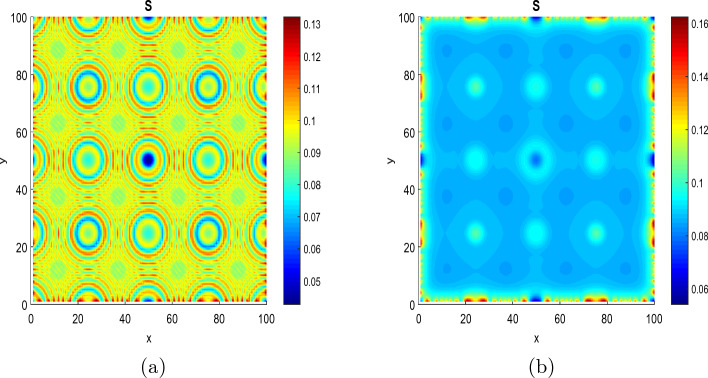
Panels show the feature of patterns at fixed parameters $\tau =\tau _{c}$ = 11.8964 and time t = 16. (**a**) $D_{S}=D_{D}=0.0031536$ and $D_{a}=D_{m}=0.0365$ and (**b**) $D_{S}=D_{D}=0.031536$ and $D_{a}=D_{m}=0.365$.

Figure 3 shows the process of pattern formation for different values of diffusion coefficients at $\tau =\tau _{c}$ = 11.8964.

The Fig. 3 exhibits the formation of circular patterns. Such circular patterns have great pre-clinical diagnostic and therapeutic potentials in multiple cancers. These have been reported playing important roles in multiple malignant behaviors including proliferation, migration, metastasis and chemoresistance^54^. Pattern formation has been also observed in other biological systems to explain energy transport in DNA strands^55,56^, impulse propagation in neural networks^56,57^ and instabilities in directed networks^58^.

In Fig. 3a the patterns are obtained for $D_{S}=D_{D}=0.0031536$ and $D_{a}=D_{m}=0.0365$. The patterns in the spatial domain represent the distribution and density of cancer cells in different regions. Since the patterns are more localized in the center, it may indicate that the cancer cells can not migrate far from their origin. This can be related to non-metastasized behavior^44^. In Fig. 3b, the patterns are obtained for the diffusion coefficients such that $D_{S}=D_{D}=0.031536$ and $D_{a}=D_{m}=0.365$ (we have increased the diffusion coefficients). We observe that the patterns are less localized in the center of the spatial domain. This may suggest that the cancer cells have dispersed widely and reached distant areas. Since metastasized behavior refers to the spread of cancer cells from their original site to other parts of the body. The scenario observed in this figure can be related to metastasized behavior. In this case, the function and structure of the surrounding tissues and organs can be affected^44^.

**Figure 4:**
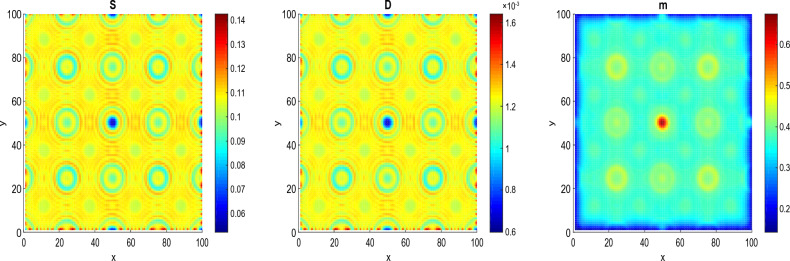
The process of formation of circular patterns for a value of $\tau =6.8964<\tau _{c}=11.8964$ and the parameters $D_{S}=D_{D}=0.0031536$, $D_{a}=D_{m}=0.0365$, at time $t=16$. The values of the other parameters are given in the Table 1.

**Figure 5:**
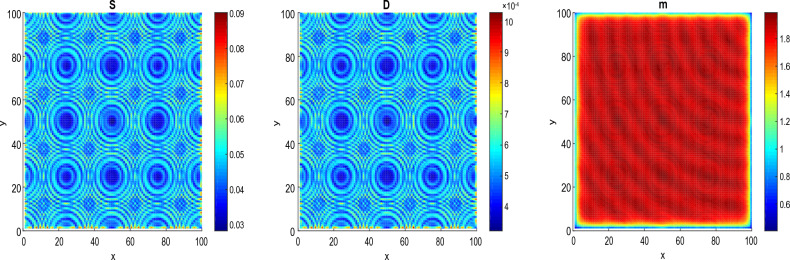
The process of formation of patterns at $\tau$ $=14.8964>\tau _{c}=11.8964$, $t=16$, $D_{S}=D_{D}=0.0031536$ and $D_{a}=D_{m}=0.0365$. The values of the other parameters are given in the Table 1.

Figure 4 shows the emergence of circular patterns in the system for the different species of the model. We assume the time delay $\tau$= 6.8964, which means 5 years before the dedifferentiation of DCs. Such circular patterns of CSCs, DCs and microRNAs obtained in this work have been observed in some solid tumors, where the CSCs surrounded by the DCs forming protective niche^59^. Circular patterns of CSCs, DCs and microRNAs obtained can be used as biomakers for diagnosis, by allowing to identify the type, stage and molecular subtype of the tumor. For example, circular pattern of microRNAs can be detected in blood, urine or biopsies of patients and can reflect the genetic profile of the tumor. Circular patterns of CSCs and DCs can be observed by imaging techniques such as positron emission tomography and can indicate the degree of aggressiveness and vascularization of the tumor^59^. In addition to diagnosis, circular patterns of CSCs, DCs and microRNAs, can also be used as biomarkers for cancer prognosis, by allowing to evaluate the risk of recurrence, progression or metastasis of tumor. For example, circular pattern of microRNAs can be associated with the overall survival or disease-free survival of patients and can predict the response to treatments^54^. Circular patterns of CSCs and DCs can be related to the invasive and metastatic potential of the tumor and can influence choice of treatment.

In Fig. 5, we assume the time $\tau$ = 14.8964, which means 3 years after the dedifferentiation of DCs. As one can observe, after the dedifferentiation, these circular patterns become more localized. The emergence of localized circular patterns can also be the result of physical, chemical or biological processes. For example, at the molecular level, circular patterns can be observed in the structure of some molecules such as circular RNAs. At the cellular level, circular patterns can be involved in the dynamics of membranes. For example the formation of curvature, fission and sorting of lipids in the membranes can be influenced by proteins that induce circular patterns. At the tissue level, circular patterns can be associated with morphogenesis and differentiation. At the organ level, circular patterns can be related to circulation and respiration^60^. Circular pattern are also important because of their prominence in virus DNA.

In addition to the fact that for the numerical point of view, colors in patterns generally represent different level of cell concentrations, from the biological point of view, the colors in circular patterns obtained in this work indicate that as genes recombine inside the cell, the cell elaborates a color unique to its genetic code. For cancer stem cells, that color becomes a genetic signature passed down to daughter cells. Blue stem cells, for example, will only make blue cancer cells. Along the same line, researchers at Harvard Medical School and Boston Children’s Hospital reported a new color-coding tool enabling scientists to better track live blood stem cells over time, a key part of understanding how blood disorders and such cancers as leukemia arise^61^.

#### Radiotherapy modeling and effects

In this section, we focus on the radiotherapy effects on the dynamical system (1). Radiation induced cell death. Radiation directly affects DNA molecule in the target tissue, single broken strand can usually be repaired by the cell while two broken strands commonly result in cell death. We know that it is possible to identify, isolate and quantify CSCs in a large number of cancers including brain, colon, prostate, lung, breast, blood^62^. Several studies^63–65^ have shown that CSCs are resistant to cytotoxic treatments such as radiotherapy. For example, numerous studies have demonstrated the high resistance of CSCs to in vitro exposure to single-dose, low LET ionizing radiation and chemotherapy. However, the effects of fractionated radiation on CSCs have not been sufficiently studied. We will examine through numerical simulations the behavior of the system when irradiated with X-rays before resurgence using fractionated doses. Radiation fractionated therapy for treatment of cancer has many advantages over single radiation administration as it raises the effect of anticancer therapy and decreases the chances of side effects occurrence in normal tissues. The dose fractionation in radiotherapy is a technique that involves dividing the total radiation dose into several smaller sessions, called fractions. This approach is used to treat cancer patients with a higher radiation dose while minimizing side effects on the surrounding normal tissues. The importance of dose fractionation in radiotherapy lies in several key aspects namely protection of normal tissues, by dividing the total dose into smaller fractions. It allows healthy tissues to recover between sessions, thus reducing undesirable side effects. This can improve treatment efficacy by causing more damage to cancer cells. Dose fractionation allows physicians to personalize treatment based on specific patient characteristics, such as tumor size and location, as well as individual tolerance to side effects. It is important to note that dose fractionation may vary depending on the type and stage of cancer, tumor location and treatment goals. We now consider $R = R(t)$ the radiotherapy function. Introducing *R* in the first equation of system (1), we obtain the following system:

12 $$\begin{aligned} \left\{ \begin{aligned} \frac{d S}{d t}&=(2p(D,a)-1)S+q(m(t-\tau ))D(t-\tau )+D_{S}\Delta S-\delta RS,\\ \frac{d D}{d t}&=2(1-p(D,a))S-(d+q(m))D+D_{D}\Delta D,\\ \frac{d a}{d t}&=a(\beta S\frac{a}{1+a}-\alpha )+D_{a}\Delta a,\\ \frac{d m}{d t}&=\gamma e^{ -S/S_{0}}-\alpha m+D_{m}\Delta m, \end{aligned} \right. \end{aligned}$$

where $\delta$ is the cancer stem cells death rate by radiation therapy^66^. In linear quadratic model, *R*(*t*) can be defined as^67,68^:

13 $$\begin{aligned} R(t)= \alpha _{s} d_{t}+2\beta _{s} d_{t}\int \limits _{0}^{t} d_{t}e^{-\mu (t-t')}dt', \end{aligned}$$

where $\alpha _{s}$ and $\beta _{s}$ are the sensitivity parameters. $\alpha _{s}$ corresponds to the cell that can not repair themselves after one radiation hit (important for hight LET radiation) and $\beta _{s}$ corresponds to cell that stop dividing after more then one radiation hit, but can repair the damage caused by radiation (important for low LET radiation), $d_{t}$ is the dose-rate at time t, $\mu$ is the repair rate constant defined as $\frac{ln(2)}{t_{1/2}}$ and $t_{1/2}$ is half-time for the repair of radiation-induced DNA damage. In fractional radiotherapy, dose is applied as fractions $d_{t}$ for intervals $t_{2n-1}< t < t_{2n}$ where *n* is the fractions per day. In practice, dose fractions should be separated by 6 hours or more to permit repair to approach completion in late-responding tissues^69^. Now, based on the studies conducted by Nilsson et al.^70^ and Powathil *et al.*^71^ on the low-dose fractionated, Eq. (13) can be written as *R*(*t*) = $R_{eff} k_{R}$ where $R_{eff}$ indicates the effect of *n* fractions per day and $K_{R}$ is a factor equal to one when the radiation is applied and is equal to zero when no radiation is applied. The effective radiation can be written as:

14 $$\begin{aligned} R(t)=R_{eff}=\alpha _{s}(n{\mathcal {D}}) + \beta _{s}n({\mathcal {D}})^{2}\biggl [g(\mu \tau _{r}) + 2\biggl (\frac{cosh(\mu \tau _{r})-1}{(\mu \tau _{r})^{2}}\biggl )h_{n}(\phi ) \biggl ], \end{aligned}$$

where $\tau _{r}$ is the duration of irradiation, ${\mathcal {D}}$ is the accumulated dose at time $\tau _{r}$ per fraction such that ${\mathcal {D}}=d_{t}\tau _{r}$ and function $\phi = e^{-\mu (\tau _{r} + \Delta \tau _{r})}$, where $\Delta \tau _{r}$ is the time interval between fractions. The functions $g(\mu \tau _{r})$ and $h_{c}(\phi )$ are defined as:

15 $$\begin{aligned}{} & {} g(\mu \tau _{r}) = 2\frac{(\mu \tau _{r}-1+e^{-\mu \tau _{r}})}{(\mu \tau _{r})^{2}}, \end{aligned}$$

16 $$\begin{aligned}{} & {} h_{n}(\phi ) = 2\frac{n\phi -n\phi ^{2}-\phi +\phi ^{n+1}}{n(1-\phi )^{2}}. \end{aligned}$$

The function $g(\mu \tau _{r})$ takes into account repair during fractions, the $h_{n}(\phi )$ function takes into account incomplete repair between fractions and $\biggl [g(\mu \tau _{r}) + 2\biggl (\frac{cosh(\mu \tau _{r})-1}{(\mu \tau _{r})^{2}}\biggl )h_{n}(\phi ) \biggl ]$ is a function that corrects for both repair during the irradiation and incomplete repair between fractions.

####  Biological effective dose (BED): determination of the optimum dose

   BED is the theoretical dose, which, if delivered in infinitely small fractions, would produce the same biological endpoint as that under consideration^72^. It is also a measure of the biological dose delivered to a tumor or organ. Treatment with any cytotoxic agent, including radiation can trigger surviving cells in tumor to divide faster than before, this phenomenon is called repopulation. Considering the proliferation of cancer stem cells during radiotherapy treatment from the LQ model^73^, $log_{e}$ tumor cell kill *E* is given by

17 $$\begin{aligned} E = \alpha _{s}n{\mathcal {D}}\biggl (1+\frac{{\mathcal {D}}}{\alpha _{s}/\beta _{s}}\biggl )\biggl [g(\mu \tau _{r}) + 2\biggl (\frac{cosh(\mu \tau _{r})-1}{(\mu \tau _{r})^{2}}\biggl )h_{n}(\phi ) \biggl ]-K(T-T_{d}), \end{aligned}$$

where *T* is the treatment time under consideration and $T_{d}$ is the time after the start of treatment at which proliferation begins. *K* (in units of *Gy*/*day*) is the biological dose per day required to compensate for ongoing cancer stem cells repopulation, once this has started. *K* is the daily BED that must be delivered simply to stop the cancer stem cells growing further during treatment. The BED for the calculation of relationships in normal tissues defined by Barendsen^74^ is given by

18 $$\begin{aligned} BED_{late} = n{\mathcal {D}}\biggl (1+\frac{{\mathcal {D}}}{\theta _{late}}\biggl ){\mathcal {M}}, \end{aligned}$$

where ${\mathcal {M}}=\biggl [g(\mu \tau _{r}) + 2\biggl (\frac{cosh(\mu \tau _{r})-1}{(\mu \tau _{r})^{2}}\biggl )h_{n}(\phi ) \biggl ]$, $\theta _{late}$ is the $\alpha _{s}/\beta _{s}$ ratio for the normal tissue late effects and ${\mathcal {D}}$ is again the accumulated dose at time $\tau _{r}$ assumed to be the same for the cancer as for the adjacent critical normal tissues which is true in many practical situations. The consideration of the repopulation cancer stem cells effect is achieved through the use of a subtractive repopulation factor which takes account of the treatment and repopulation rate, the expression of BED is

19 $$\begin{aligned} BED_{can} = n{\mathcal {D}}\biggl (1+\frac{{\mathcal {D}}}{\theta _{can}}\biggl ){\mathcal {M}}-K(T-T_{d}), \end{aligned}$$

where $\theta _{can}$ is the $\alpha _{s}/\beta _{s}$ ratio for the cancer stem cells effects.

The relationship between *n* and *T* can be approximated as

20 $$\begin{aligned} T=fn-1, \end{aligned}$$

where *f* approximates to 7/5 for 5 fractions per week, providing more than 5 fractions are given^75^. This form of approximation becomes more accurate when *T* is relatively large (greater than 14 days). Eq. (19) can be rewritten as

21 $$\begin{aligned} BED_{can}=\biggl (\frac{BED_{late}}{1+{\mathcal {D}}/\theta _{late}}\biggl ) \biggl (1+\frac{{\mathcal {D}}}{\theta _{can}}\biggl )-K\biggl (f\frac{BED_{late}}{{\mathcal {D}}(1+{\mathcal {D}}/\theta _{late}){\mathcal {M}}}-1-T_{d}\biggl ). \end{aligned}$$

When the terms are rearranged and $BED_{can}$ is differentiated with respect to ${\mathcal {D}}$, a dose per fraction given by the solution of

22 $$\begin{aligned} \biggl (1-\frac{\theta _{late}}{\theta _{can}}\biggl ){\mathcal {M}}{\mathcal {D}}^{2}-2Kf{\mathcal {D}}-Kf\theta _{late}=0. \end{aligned}$$

The positive root of this quadratic equation provides the value of the optimum dose per fraction:

23 $$\begin{aligned} {\mathcal {D}}{opt}=\frac{Kf+\sqrt{K^{2}f^{2}+(1-\theta _{late}/\theta _{can}) Kf\theta _{late}}}{(1-\theta _{late}/\theta _{can}){\mathcal {M}}}. \end{aligned}$$

We calculate the BED using the values of parameters in Table 2 and the values of radiobiological parameters for prostate cancer: $\theta _{late}=3$ Gy, $T_{d}$=40 days, $K=0.2$ Gy/day^76^ and *f* = 7/5^75^. For a treatment time T = 45 days with 2 dose fractions per day, we obtain the values of the optimal dose and the BED given by

24 $$\begin{aligned} {\mathcal {D}}{opt}= 1.328 \text{Gy} \;\ \text{and} \;\ BED_{can}= 4.097 \text{Gy} \end{aligned}$$

respectively.

#### The tumor control probability (TCP)

The TCP is a key indicator to assess the effectiveness of cancer treatment^77^. A higher TCP is generally associated with better outcomes and longer survival for patients. However, there are several factors that can influence the TCP. The stage of cancer is one of these important factors, as indicates the extent of the disease. Early-stage tumors typically have a higher TCP than advanced-stage tumors. The location of tumor is also a factor to consider, as some tumors may be more challenging to treat due to their location in sensitive or hard-to-reach areas. The type of treatment used also plays a key role. Different types of treatments, such as surgery, radiotherapy and chemotherapy may have different effects on the tumor control probability. The patient’s response to treatment is also an important factor. Each patient responds differently to treatments and how the body reacts can influence the tumor control probability. The (TCP) is the probability of avoiding local recurrence at total dose $n{\mathcal {D}}$^78^. The Poisson’s statistics and the linear-quadratic model incorporating the Poisson’s law^79^ lead to

25 $$\begin{aligned} TCP=exp(-N.S_{v})=exp(-Nexp(-\alpha _{s}BED_{can})), \end{aligned}$$

where $N=\varsigma .V$ ( $\varsigma$ is cell concentration or density and *V* is volume) represents the initial number of potential proliferating cells in tumor and $S_{v}$ is the cell survival probability such that $S_{v}=exp(-\alpha _{s}BED_{can})$. The closer TCP is to one, the greater the probability that all cancer stem cells die out and the tumor is controlled^80^. Spoormans et al.^81^ discussed that TCP takes into account the total absorbed dose and the radiosensitivity of the tumour tissue, while distinguishing between repairable and sublethal single-strand DNA breaks and irreparable and lethal double-strand DNA breaks. The parameters related to radiotherapeutic values are given in the Table 3.

**Table 3:** Values of the radiobiological parameters.

Parameter	Value/unit	References
$\alpha _{s}$	$0.302 \textrm{Gy}^{-1}$	^82^
$\beta _{s}$	$0.0417 \textrm{Gy}^{-2}$	^82^
$\delta$	$25\, \textrm{year}^{-1}\textrm{Gy}^{-1}$	^66^
$\mu$	$3195.77 \textrm{year}^{-1}$	^83^

**Figure 6:**
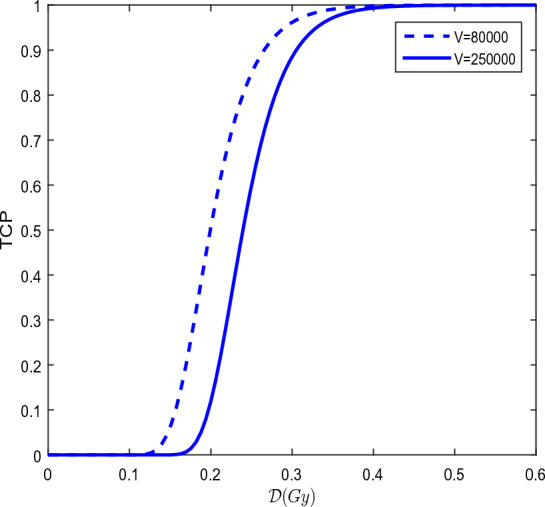
Evolution of TCP as a function of fractionated dose ${\mathcal {D}}$ for a treatment time T= 45 days, with 2 dose fractions per day when the prostate tumor volume is 80000 $\textrm{mm}^{3}$(dashed line) and when the prostate tumor volume is 250000 $\textrm{mm}^{3}$ (solid line). That means the radiotherapy is applied on five successive days per week.

Figure 6 shows the TCP as a function of fractionated dose ${\mathcal {D}}$ when the concentration *S* of cancer stem cells is equal to 0.000805 $\mathrm{cell.mm}^{-3}$ at the starting time of the radiotherapy and for a prostate tumor that measures $8 \textrm{mm}$ visible on magnetic resonance imaging^84^. The volume of the tumor in the area $(100 \,\textrm{mm}\times 100 \,\textrm{mm})$ is 80000 $\textrm{mm}^{3}$ and when the prostate tumor measures 25 $\textrm{mm}$. The values of the parameters in the Table 3.

Figure 6 shows that the 2 dose fractions per day option requires 0.5273 $\textrm{Gy}$ to avoid possible local resurgence of cancer after radiotherapy when tumor measures 8 $\textrm{mm}$. This requires 0.5697 $\textrm{Gy}$ to avoid local resurgence after radiotherapy when tumor measures 25 $\textrm{mm}$.

#### Radiotherapy effects on the time series

**Figure 7:**
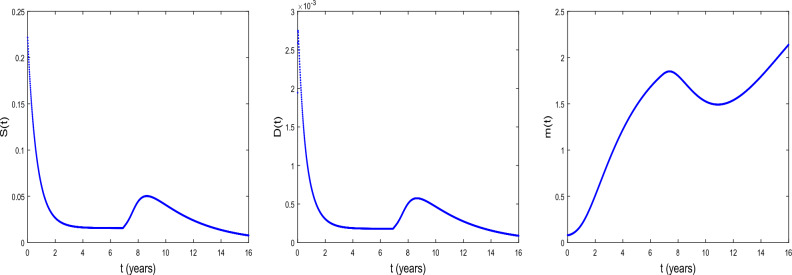
Time series of *S*, *D* and *m* under radiotherapy at $\tau =6.8964 <\tau _{c}=11.8964$, $t=16$. $D_{S}=D_{D}=0.0031536$ and $D_{a}=D_{m}=0.036500$. ${\mathcal {D}}$ = 0.02 Gy $, n= 2$, $\tau _{r}$ =0.000009 year $\sim$ 5 min and $\Delta \tau _{r}$ = 0.00069 year $\sim$ 6.04 h. The values of the other parameters are given in the Table 3.

**Figure 8:**
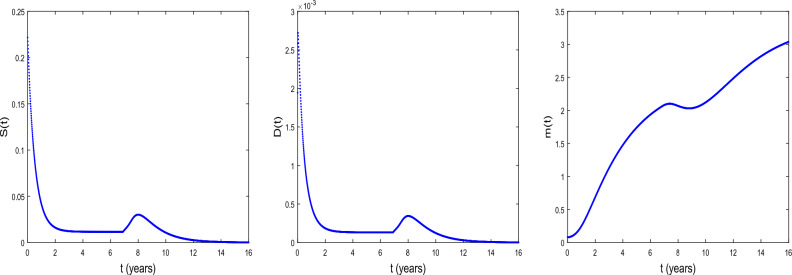
Time series of *S*, *D* and *m* under radiotherapy at $\tau =6.8964<\tau _{c}=11.8964$, $t=16$. $D_{S}=D_{D}=0.0031536$ and $D_{a}=D_{m}=0.036500$. ${\mathcal {D}}$ = 0.05 Gy, $n= 2$, $\tau _{r}$ =0.000009 year $\sim$ 5 min and $\Delta \tau _{r}$ = 0.00069 year $\sim$ 6.04 h. The values of the other parameters are given in the Table 3.

**Figure 9:**
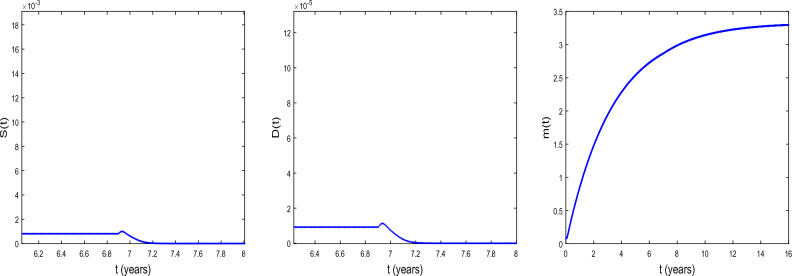
Time series of *S*, *D* and *m* under radiotherapy at $\tau =6.8964<\tau _{c}=11.8964$, $t=16$. $D_{S}=D_{D}=0.0031536$ and $D_{a}=D_{m}=0.036500$. ${\mathcal {D}}$ = 1.323 Gy, $n= 2$, $\tau _{r}$ =0.000009 year $\sim$ 5 min and $\Delta \tau _{r}$ = 0.00069 year $\sim$ 6.04 h. The values of the other parameters are given in the Table 3.

Radiotherapy is one of the treatment modalities being substantially benefited in cancer research. Figures 7, 8 and 9 show the time series of *S*, *D* and *m* under the radiotherapy effects. The radiotherapy is applied at the time $t=6.8964$ before the critical time delay $\tau _{c}=11.8964$. The figures illustrate the changes in the concentration of prostate CSCs, DCs and microRNAs under the radiotherapy effects. We observe that the concentrations of CSCs and DCs decrease progressively with an increasing of the fractional dose. In contrast, the concentration of microRNAs increases when the fractional dose increases. This reflects the over-expression of microRNA under the radiotherapy effects as also found in^85^. Along the same line, it has been shown that in prostate cancer patients, two microRNAs hsa-let-7a-5p and hsa-miR-21a-5p, were regulated by irradiation^86^. Similarly, Halimi et al.^87^ showed that irradiation during radiotherapy increases the serum miR-34a levels in 44 women with breast cancer. The obtained figures show that radiotherapy effects modify the expression of microRNAs and lead the CSCs to be radiosensitive to radiation. We also observe in these figures a tumor repopulation during radiotherapy. This confirm the results obtained in^88^, where the investigators revealed that rapid tumor regrowth occurs during radiotherapy treatment extensions of around 5 to 8 weeks in almost 500 patients with oropharyngeal cancer^88^. It is therefore necessary to administer a higher dose to regions of tumor repopulation for complete eradication of CSCs. In the present work, the CSCs are completely eradicated when the treatment lasts 0.3 year ($\sim$ 3.7) months for a dose of 1.323 $\textrm{Gy}$.

#### Radiotherapy effects on the pattern formation

**Figure 10:**
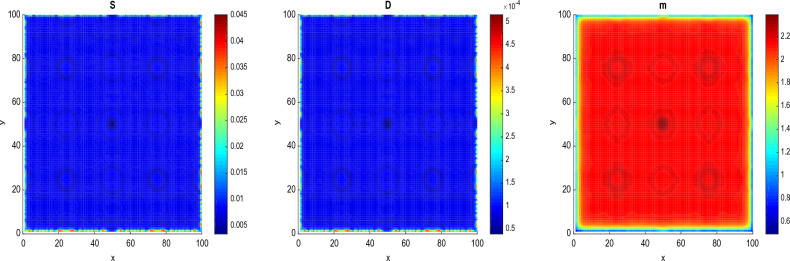
Behavior of *S*, *D* and *m* patterns under the radiotherapy at $\tau =6.8964<\tau _{c}=11.8964$, $t=16$. $D_{S}=D_{D}=0.0031536$ and $D_{a}=D_{m}=0.036500$. ${\mathcal {D}}$ = 0.02 Gy, $n= 2$, $\tau _{r}$ = 0.000009 year $\sim$ 5 min and $\Delta \tau _{r}$ = 0.00069 year $\sim$ 6.04 h. The values of the other parameters are given in the Tables 1 and 3.

**Figure 11:**
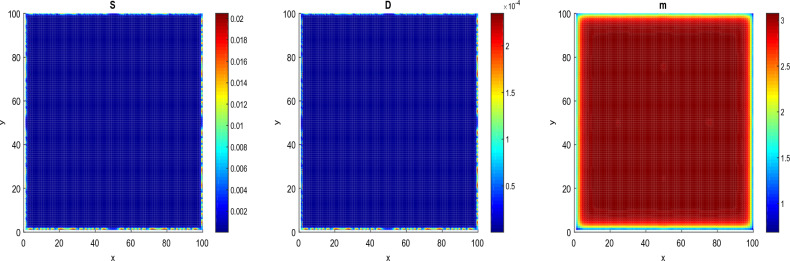
Behavior of *S*, *D* and *m* patterns under the radiotherapy at $\tau =6.8964<\tau _{c}=11.8964$, $t=16$. $D_{S}=D_{D}=0.0031536$ and $D_{a}=D_{m}=0.036500$. ${\mathcal {D}}$= 0.05 Gy, $n= 2$, $\tau _{r}$=0.000009 year $\sim$ 5 min and $\Delta \tau _{r}$= 0.00069 year $\sim$ 6.04 h. The values of the other parameters are given in the Tables1 and 3.

This section is devoted to the radiotherapy effects on the patterns of cancer cell colony. To this end, we perform extensive numerical simulations of the mathematical model Eq. (12). The radiotherapy is applied at the time $t=6.8964>\tau _{c}=11.8964$ before the critical time delay that is in the non-proliferation state of CSCs. Figures 10 and 11 illustrate the radiotherapy effects on the spatiotemporal dynamics of patterns. These figures show the changes in the prostate CSCs, DCs and microRNAs patterns when the dose of fractionation radiotherapy varies. In Fig. 10, we use the radiobiological values ${\mathcal {D}} = 0.02 \,\textrm{Gy}$, $n= 2$, $\tau _{r} =0.000009$ year $\sim 5$ min and $\Delta \tau _{r}= 0.00069$ year $\sim 6.04$ h. We observe a partial eradication of patterns of CCSs and DCs and a decrease in the concentration level of cells as compared with Figs. 3 and 4. However, we observe an increase of the concentration level on the patterns of microRNAs leading to the fact that they can act as suppressor in certain tumors. In Fig. 11, we take ${\mathcal {D}} = 0.05\, \textrm{Gy}$, that is, we increase the fractionated dose. In this case, the panels show a complete eradication of patterns of CCSs and DCs. The result obtained in this section clearly demonstrates that the administering of a therapeutic treatment, such as the radiotherapy, before the critical time delay $\tau _{c}$, can lead to a complete eradication of the tumor.

## Discussion and conclusion

In this paper, we studied a spatiotemporal dynamics of a diffusive model for CSCs. The model included the diffusion of CSCs, DCs, Wnt-$\beta$ and microRNA molecules. The work focused on the critical time delay $\tau _{c}$, since its represents the time from which some DCs transform into CSCs due to their interaction with microRNAs. We showed that the delayed model can induce a Hopf bifurcation and circular pattern formation. We have found that $\tau _{c}$ = 11.8964 years for prostate cancer, $\tau _{c}$ = 0.6884 year for lung cancer and $\tau _{c}$ = 0.5763 year for breast cancer. As therapeutic strategy, radiotherapy effects have been included in the model and applied before $\tau _{c}$. The results revealed that radiotherapy effects can completely eradicate cancer patterns and boost the suppressive effect of microRNAs on CSCs. The TCP curve showed that for the prostate tumor volumes equal to 80000 $\textrm{mm}^{3}$ and 250000 $\textrm{mm}^{3}$, for example, the respective maximum doses of 0.5354 $\textrm{Gy}$ and 0.5758 $\textrm{Gy}$ per dose fraction should be administered for a fractionation of 2 fractions per day. The results clearly illustrated that any resurgence of cancer at the same site after radiotherapy is not possible for a fraction of the dose required.

Before closing this work, it is important to point out the essential new features of this study with respect to some recent works for CSCs available in the literature^18–20^. To start with, in^18^, the authors used a concept from ecology, the Allee effect, to demonstrate the effectiveness of combination therapies in cancer treatment compared to mono-therapy. In^19^, the authors studied clonal pattern dynamics in tumor in the concept of CSCs and showed that the most effective therapeutic strategies for tumor extinction are those that target chemical activators rather than cells themselves, due to a vicious feedback loop between CSCs and DCs. In^20^, the authors investigated the dynamical model for CSCs taking into account the drug effects and showed some interesting mechanism, such as the tumor growth paradox, as possible out come of therapy significantly. However, the works cited above and some other implicitly assume that biological transitions such as interactions between cancer cells with signaling molecules and proteins are instantaneous. In reality, the time delay exists between such interactions. This is important because DCs interact with microRNAs to transform into CSCs. In the present work, the proposed basic model illustrated this type of scenario. We showed that a diffusive model of CSCs with a time delay can be an effective modeling approach for therapeutic strategies. With the advancement of novel technology and methods, the circular patterns obtained numerically in this work have been found to give relevance biological significance. Just to cite a few, circular patterns have been identified to be extremely abundant, relatively stable, diverse, prevalent, and conserved in different diseases^89^. Circular RNAs may aggregate in cells to induce pathologies, such as developmental and degenerative disorders^90^. In addition, circular RNAs are expressed in a tissue-specific pattern, suggesting that they act as promising diagnostic and therapeutic value in management of cancer and various diseases^76,91,92^. We clearly showed that the administering of a therapeutic treatment, such as the radiotherapy, before the critical time delay $\tau _{c}$ (that is during the non-proliferation phase of CSCs), can lead to a complete eradication of the tumor. This approach has the virtue of describing therapeutic strategies of the dynamics of CSCs with time delay. From that perspective, a wide range of phenomena in biology, physics and chemistry can be modeled by the present diffusive delayed system, and relevant applications to these areas may be proposed.

We hope that the results obtained in this work can provide a better understanding of the dynamics of CSCs and to predict more efficacy therapeutic strategies to prevent cancer recurrence.

### Supplementary Information

Supplementary Information.

## Data Availability

All data generated or analyzed during this study are included in this paper.
