# Supplementary material for: Dynamics of a diffusive model for cancer stem cells with time delay in microRNA-differentiated cancer cell interactions and radiotherapy effects
Source: Sci Rep. 2024 Mar 4;14:5295. doi: 10.1038/s41598-024-55212-4 (PMC10912232; doi:10.1038/s41598-024-55212-4)
Supplement: Supplementary file 1 — Supplementary Information. [file 41598_2024_55212_MOESM1_ESM.pdf]

Appendix

$$
\begin{aligned}
C_{11} =& k^2(D_D + D_a + D_m + D_S) + 2d + 2\alpha + 1,
\end{aligned}
$$

$$
\begin{aligned}
C_{22} =& k^4(D_D D_a + D_D D_m + D_D D_S + D_a D_m + D_a D_S + D_m D_S) \\
&+ k^2(2dD_a + 2dD_m + 2dD_S + 2\alpha D_D) \\
&+ k^2(\alpha D_a + \alpha D_m + 2\alpha D_S + D_D + D_a + D_m) \\
&+ 4d\alpha + \alpha^2 + 2d + 2\alpha,
\end{aligned}
$$

$$
\begin{aligned}
C_{33} =& k^6(D_D D_a D_m + D_D D_a D_S + D_D D_m D_S + D_a D_m D_S) + 2dk^4(D_a D_m + D_a D_S + D_m D_S) \\
&+ k^4(\alpha D_D D_a + \alpha D_D D_m + 2\alpha D_D D_S + \alpha D_a D_S + \alpha D_m D_S) \\
&+ k^4(D_D D_a + D_D D_m + D_a D_m) \\
&+ k^2(2d\alpha D_a + 2d\alpha D_m - 4d\alpha D_S + \alpha^2 D_D + \alpha^2 D_S + 2dD_a + 2dD_m + 2\alpha D_D + \alpha D_a + \alpha D_m) \\
&+ 2d\alpha^2 + 4d\alpha + \alpha^2,
\end{aligned}
$$

$$
\begin{aligned}
C_{44} =& k^8 D_D D_a D_m D_S + k^2(2dD_a D_m D_S + \alpha D_D D_a D_S + \alpha D_D D_m D_S + D_D D_a D_m) \\
&+ k^4(2d\alpha D_a D_S + 2d\alpha D_m D_S + \alpha^2 D_D D_S + 2dD_a D_m + \alpha D_D D_a + \alpha D_D D_m) \\
&+ k^2(2d\alpha^2 D_S + 2d\alpha D_a + 2d\alpha D_m + \alpha^2 D_D) \\
&+ 2d\alpha^2,
\end{aligned}
$$

$$
E_{11} = -\frac{1}{\sigma S_0 q_0}\left[2\alpha d^2 D_2 m_2 + 2d\sigma S_0 q_0 - 2\alpha d D_2 m_2 q_0\right],
$$

$$
\begin{aligned}
E_{22} = -\frac{1}{\sigma S_0 q_0}\Big[& k^2(2\alpha d^2 D_2 D_D m_2 + 2\alpha d^2 D_2 D_a m_2) + k^2(2d\sigma D_a S_0 q_0 + 2d\sigma D_m S_0 q_0) \\
&+ k^2(-2\alpha d D_2 D_D m_2 q_0 - 2\alpha d D_2 D_a m_2 q_0) + 4\alpha d^3 D_2 m_2 + 2\alpha^2 d^2 D_2 m_2 - 4\alpha d^2 D_2 m_2 q_0 \\
&+ 4d\alpha\sigma S_0 q_0 - 2\alpha^2 d D_2 m_2 q_0 - 2\alpha d^3 D_2 m_2 + 2\alpha d^2 D_2 m_2 q_0\Big],
\end{aligned}
$$

$$
\begin{aligned}
E_{33} = -\frac{1}{\sigma S_0 q_0}\Big[& k^4(2\alpha d^2 D_2 D_D D_a m_2 + 2d\sigma D_a D_m S_0 q_0 - 2\alpha d D_2 D_D D_a m_2 q_0) \\
&+ k^2(4\alpha d^3 D_2 D_a m_2 + 2\alpha^2 d^2 D_2 D_D m_2 - 4\alpha d^2 D_2 D_a m_2 q_0 + 2d\alpha\sigma D_a S_0 q_0 \\
&+ 2d\alpha\sigma D_m S_0 q_0 - 2\alpha^2 d D_2 D_D m_2 q_0 - 2\alpha d^3 D_a D_2 m_2 + 2\alpha d^2 D_a D_2 m_2 q_0) \\
&+ 4\alpha^2 d^3 D_2 m_2 - 4\alpha^2 d^2 D_2 m_2 q_0 + 2d\alpha^2\sigma S_0 q_0 - 2\alpha^2 d^3 D_2 m_2 + 2\alpha^2 d^2 D_2 m_2 q_0\Big].
\end{aligned}
$$
